# 5 Tips for Residents New to Teaching Medical Students

**DOI:** 10.15694/mep.2017.000057

**Published:** 2017-03-20

**Authors:** Cyril Eyadiel, Charles Randall Clinch

**Affiliations:** 1 Wake Forest School of Medicine Family Practice Residency Program; 2 Wake Forest School of Medicine

**Keywords:** resident as teacher, medical student, medical training

## Abstract

This article was migrated. The article was marked as recommended.

Graduating from medical school is a time of transition that is filled with many responsibilities and opportunities. In a few short years, new physicians are expected to learn their trade and teach what they have learned to students along the way before being allowed to practice independently. As a medical student, trainees have the opportunity to learn from multiple providers from a range of specialties at all levels of training. Physicians in graduate medication education programs (i.e., residents) have a unique opportunity to provide training and teaching to medical students with a perspective that comes from being between the student and the expert attending physician. Whether students work with a resident during one day or over the course of several weeks, this time can be especially helpful in growing the student’s knowledge base while honing the resident’s teaching skills. The purpose of this article is to provide tips for resident physicians to use as they begin their role as teachers of medical students while balancing their responsibilities as trainees themselves. Many residency programs are developing formal resident as teacher programs to help facilitate their residents in becoming better teachers. These tips may be applied in such programs.

## Introduction

A universal aspect of medicine is the role of the physician as teacher. Physicians must explain a patient’s condition to them in an understandable manner within a limited period of time. This role as teacher does not come naturally to all who enter the profession of medicine. For some, teaching is a frustrating responsibility. This can be especially true for a resident physician who is tasked with the steep learning curve of understanding disease processes, the increased responsibility for medical decision making, and teaching patients while training the next generation of physicians - medical students. Teaching medical students is, however, an opportunity that allows the resident physician to challenge their own understanding of disease processes. The following tips may make the experience of teaching medical students more rewarding for residents and more effective for students.

## Tip 1: Set the tone

Whether your interaction with a medical student is for a few hours or a few weeks, it is essential to establish some ground rules and to determine what the student is particularly interested in learning. Start by getting to know the student on a personal level and demonstrate that you are approachable. ^1^ This may reduce anxiety in the student and result in better performance (e.g., more organized presentations). Ground rules can include when to arrive, what work is needed to complete prior to the start of the day, when to ask questions during a clinic or on rounds, and how long to spend interacting with a patient. Ask what the student wants to get out of the particular encounter or rotation. This will help establish a focus while targeting the student’s particular learning needs. If this time is invested on the front end, both parties will be more satisfied as expectations are well known and established for the experience.

## Tip 2: Ask “What if…?”

Resident physicians may recall instances when attending physicians have asked, “What if..?”. It is an important question to raise if we want to help develop medical students to become thinkers. Students often present their patients with a limited focus and thus narrow differential diagnoses and treatment plans. This may relate to the breadth of the student’s knowledge base, which should expand with time. Alternatively, the student may possess the knowledge yet lack the experience to direct their diagnostic considerations or treatment options. In this case, the resident physician can use their knowledge and experience to ask what if another symptom presented or the history/physical exam was different. Such questioning will allow the student to demonstrate their ability to widen their diagnostic or treatment considerations without having to lead them. This approach can simulate having multiple patient encounters though the student only interacted with one patient.

## Tip 3: Have patience

With the growing demands on a resident physician’s time, demonstrating patience is even more important, both with patients and with medical students. Most residents can recall a time when their case presentations were cut short due to their teacher’s impatience. A key characteristic of a good teacher is patience. Allow students to complete their thoughts without interruption and then give them time to respond to questions before supplying them with hints or responding on their behalf. A good rule of thumb is giving students 3 minutes to present their patient before interrupting them for more details. Establish this expectation when covering your ground rules. Given a distinct time boundary, medical students can learn to become quicker and more efficient with their presentations. ^3^

## Tip 4: Highlight key points in the patient presentation that leads to the diagnosis

Highlighting the key points in a patient presentation that leads to an accurate diagnosis will help reinforce the case for a medical student. ^3^ Additionally, this allows them to understand your thought process and redirect their focus in a patient encounter, helping them come to the diagnosis. Summarizing the information gathered and reframing the full picture concisely will help solidify the diagnosis and treatment plan. Students are initially trained to be information gatherers; processing information and formulating a plan are skills taught as they progress through the clinical years. How efficiently and effectively this is learned is partially student dependent and is certainly affected by their clinical teachers. Repeating key points and reiterating the most salient facts will help the student reflect and hone in on the most important parts of the patient’s story that leads to the appropriate diagnosis. ^2^ When experiencing a similar situation in the future, the medical student will ideally remember the approach that was applied.

## Tip 5: Provide specific feedback

Residents should provide specific feedback to medical students as early as possible in the interaction. This will prevent students from repeating the same errors throughout the duration of the clinic day or rotation. In addition, providing specific feedback in the moment will allow the student to relate the feedback to a specific instance which makes it more salient and memorable. Generally speaking, students appreciate when residents take the time to provide feedback despite the sting of receiving it in the moment. Providing positive feedback about behaviors students should repeat might be delivered in the presence of the patient or other team members. Providing negative feedback is typically best delivered on-on-one and in private. Some students need to be told that the feedback is indeed feedback for improvement and not solely related to an evaluation (e.g., “Next time you encounter this, I recommend that you..”; “To take this to the next level, I’d recommend..”). ^2^ Teachers and mentors who provide this kind of feedback are those students remember positively as helping shape them into the physician they want to become.

## Conclusion

Though the role of teacher may appear to be an extra duty on the mountain of responsibilities that rest on the shoulders of every resident, it is a valuable one that will benefit both the resident and their students. Through setting the tone, asking “What if..”, having patience, highlighting key points, and providing specific feedback, a resident can be a more effective and efficient teacher. There are many more strategies and techniques residents can use to become more effective teachers. Each resident will develop his or her own style of teaching they find works best with their work flow. Each learner is also different and teaching styles may need to be adjusted to accommodate the student. Resident physicians provide a significant amount of teaching to medical students and applying these tips can make the experience beneficial to all involved.

## Take Home Messages

• Set the tone: establish ground rules; learn something about your student; be approachable; establish some learning objectives
• Ask, “What if..”: help students become thinkers; explore what if another symptom was present or if the history/exam was different
• Have patience: give students 3 minutes to present their patient before interrupting for more details
• Highlight key points in the patient presentation that leads to the diagnosis: reinforces the case; makes your thought process explicit
• Provide specific feedback: reinforces positive behaviors that should be repeated; helps students avoid repeating undesired behaviors

## Notes On Contributors

Cyril Eyadiel, MD, is a third-year resident in Family Medicine at the Wake Forest School of Medicine Family Medicine Residency Program.

Charles Randall Clinch, DO, MS, is associate dean for academic accreditation and associate professor, Family & Community Medicine, Wake Forest School of Medicine.
