# Supplementary material for: 5 Tips for Residents New to Teaching Medical Students
Source: MedEdPublish (2016). 2017 Mar 20;6:57. [Version 1] doi: 10.15694/mep.2017.000057 (PMC10885247; doi:10.15694/mep.2017.000057)
Supplement: Peer review history

# Reviewer report: Reviewer response for version 1

**DOI:** 10.21956/mep.19467.r28703

**Published:** 2018-06-08

**Authors:** Anthony David.M

**Affiliations:** 1 Soho

This review has been migrated. The reviewer awarded 4 stars out of 5 An enjoyable paper indeed. I remember how my knees knocked when I started my first class for medical students! These five tips are very practical and I wish I had access to these before I began teaching a few decades ago.The first tip is very important as it allays anxiety in the participant. All the tips are 'do-able' and can be adapted in different settings. Kudos to the authors!

Reviewer Expertise:

NA

No decision status is available

# Reviewer report: Reviewer response for version 1

**DOI:** 10.21956/mep.19467.r28711

**Published:** 2017-06-01

**Authors:** Susmita Reddy Karri

**Affiliations:** 1 Topiwala national medical college and BYL nair charitable hospital, mumbai. india

This review has been migrated. The reviewer awarded 3 stars out of 5 A good paper. These tips hold true not only for residents but for all doctors. After all, the literal meaning of the word 'doctor' is 'to teach.'However, the 3 minute suggestion is a bit too far-fetch, especially in under-developed and developing countries where the patient load is too high.

Reviewer Expertise:

NA

No decision status is available

# Reviewer report: Reviewer response for version 1

**DOI:** 10.21956/mep.19467.r28704

**Published:** 2017-06-01

**Authors:** Ricardo Leon-Borquez

**Affiliations:** 1 Panamerican Federetion of Associations of Medical Schools

This review has been migrated. The reviewer awarded 2 stars out of 5 Nice paper, very basic for teaching medical students. I liked the part in which “a resident teaching a medical student challenge their own understanding of disease processes.” I believe that this is probably the most important message for new residents teaching medical students.I also think that the resident that is going to be responsible of teaching should have the outcomes that the medical school wants for their students, since most students don’t really know “what wants to get out of the particular encounter or rotation.”The “What if…?” will allow the student to demonstrate their ability to widen their diagnostic or treatment considerations without having to lead them, but also can lead students to frustration, especially if the resident is a little pushy.The recommendation of patience is very good, but very hard to deal in a competitive residency program. Tree minutes without interruption may be too long, especially if the resident is aware that the student in minute one is saying inadequate things.Finally, the feedback is essential for improvement and is well presented, but unfortunately with misspell.

Reviewer Expertise:

NA

No decision status is available

# Reviewer report: Reviewer response for version 1

**DOI:** 10.21956/mep.19467.r28709

**Published:** 2017-05-20

**Authors:** Neel Sharma

**Affiliations:** 1 Albert Einstein College of Medicine, Montefiore Medical Center

This review has been migrated. The reviewer awarded 4 stars out of 5 Having had the experience of teaching US students physical exam techniques as a UK trained clinician I value such an article. I contemplated for some time how to engage with learners from different cultures and find the universal rule of transparency helps a great deal. I always like to reveal the mistakes I made. It is realistic and learners appreciate it. Not all faculty are keen to be transparent though

Reviewer Expertise:

NA

No decision status is available

# Reviewer report: Reviewer response for version 1

**DOI:** 10.21956/mep.19467.r28708

**Published:** 2017-04-03

**Authors:** James Fraser

**Affiliations:** 1 Griffith University

This review has been migrated. The reviewer awarded 3 stars out of 5 The authors 5 tips add to the published literature on skills for clinician teachers. It would be important to link the student’s aims for the learning opportunity to the curriculum and the student’s previous learning in the area. Knowledge of the curriculum and expected standards would also add value to the meaningfulness of the feedback provided. I liked the inclusion of having patience with learner despite the busy clinical environment; this contributes to the safety of learning environment for the student and the three minute suggestion models good listening skills to the student.

Reviewer Expertise:

NA

No decision status is available

# Reviewer report: Reviewer response for version 1

**DOI:** 10.21956/mep.19467.r28707

**Published:** 2017-03-21

**Authors:** Trevor Gibbs

**Affiliations:** 1 AMEE

This review has been migrated. The reviewer awarded 3 stars out of 5 An interesting paper that addresses some important points related to faculty teaching. There were perhaps a few areas that I would have hoped had come up in discussion:-Is faculty clinical teaching a mix of opportunistic well constructed teaching- perhaps using the 1 minute preceptor model- or structured around the agreed learning outcomes. One worry is that there will be repetitive subject teaching using the opportunistic model and frequently a missed learning objective- Should these sessions be mapped onto a curriculum map- I think that the authors night mean faculty rather than just residents- a word not recognised throughout the world and one which produces a more specific paper

Reviewer Expertise:

NA

No decision status is available

# Reviewer report: Reviewer response for version 1

**DOI:** 10.21956/mep.19467.r28710

**Published:** 2017-03-21

**Authors:** sathyanarayanan varadarajan

**Affiliations:** 1 SRM Medical College, SRM University

This review has been migrated. The reviewer awarded 4 stars out of 5 In this interesting article, the authors give five useful tips for Residents new to teaching medical students. These important tips are practical to apply and easy to follow during routine clinical teaching. These tips may be applied to help not only residents in becoming better teachers but also for other clinical teachers to excel in their teaching. The authors after explaining the five tips conclude that, through setting the tone (tip 1), asking “What if…” (tip2), having patience (tip 3), highlighting key points (tip 4), and providing specific feedback (tip 5), a resident can be a more effective and efficient teacher. The above tips can make the experience of teaching medical students more rewarding for clinical teachers and more effective for students.

Reviewer Expertise:

NA

No decision status is available

# Reviewer report: Reviewer response for version 1

**DOI:** 10.21956/mep.19467.r28706

**Published:** 2017-03-20

**Authors:** Richard Hays

**Affiliations:** 1 James Cook University

This review has been migrated. The reviewer awarded 3 stars out of 5 I enjoyed reading this paper, although it may be mis-named. It is really 'Tips for teachers of medical students: the learner' perspective'. It is therefore not so much a complete package about teaching medical students, but rather an addendum to other useful papers on the same theme. I particularly like the pleas for patience, as one role of teachers is to build confidence through allowing and providing feedback on learners' presentations and clinical reasoning.

Reviewer Expertise:

NA

No decision status is available

# Reviewer report: Reviewer response for version 1

**DOI:** 10.21956/mep.19467.r28705

**Published:** 2017-03-20

**Authors:** John Cookson

**Affiliations:** 1 University of Worcester

This review has been migrated. The reviewer awarded 4 stars out of 5 I enjoyed reading this; it's potentially a very large topic but your tips are all worthwhile. In particular I like the inclusion of what the student wants to get out of the session. Related to this is the need for the teacher to know something about what is in the curriculum. Also important is to find out what the student knows already so you can build on it.

Reviewer Expertise:

NA

No decision status is available
